# Prevalence of prolonged grief disorder in a sample of female refugees

**DOI:** 10.1186/s12888-019-2136-1

**Published:** 2019-05-14

**Authors:** Regina Steil, Jana Gutermann, Octavia Harrison, Annabelle Starck, Laura Schwartzkopff, Meryam Schouler-Ocak, Ulrich Stangier

**Affiliations:** 1 Department of Clinical Psychology and Psychotherapy, Institute of Psychology, Goethe University Frankfurt, Varrentrappstr. 40-42, 60486 Frankfurt on Main, Hesse Germany; 2 Department of Psychiatry, Institute of Medicine, University Clinic of Charité at St. Hedwig Hospital, Berlin, Germany

**Keywords:** Refugees, Asylum seekers, Prolonged grief disorder, Prevalence

## Abstract

**Background:**

Prolonged Grief Disorder (PGD) is a distinct syndrome that follows bereavement. It is different from other mental disorders and is characterized by symptoms such as yearning for the bereaved, or intense emotional pain or distress. Violent loss is one major risk factor for the development of PGD.

**Objectives:**

PGD has been studied in different populations, mostly in small samples, with only a few of them being representative. Although research highlighted that traumatic experiences paired with challenges related to migration make refugees particularly vulnerable to PGD, PGD has only rarely been studied in refugees. Thus, this article a) examines the prevalence of PGD in female refugees in Germany according to the criteria proposed by Prigerson and colleagues in 2009, and b) associates PGD with other common psychopathology (e.g. anxiety, depression, somatization and trauma).

**Method:**

A total of 106 female refugees were assessed for bereavement and PGD. Of these 106 individuals, 85 were interviewed using the Prolonged Grief Disorder Scale (PG-13). Symptoms of anxiety and depression were assessed by the Hopkins Symptom Checklist-25 (HSCL-25), somatization was assessed by the Somatization Subscale of the Symptom-Checklist-90 (SCL-90), and the number of witnessed and experienced trauma was assessed by the *Posttraumatic Diagnostic Scale* (PDS/HTQ).

**Results:**

Ninety of the 106 participants had experienced bereavement, and among those, 9.41% met criteria for PGD. The most frequent PGD symptoms were bitterness, longing or yearning for the bereaved, and lack of acceptance of the loss. Furthermore, grief symptoms were significantly associated with symptoms of depression, anxiety, somatization, and the number of experienced traumatic events.

**Conclusion:**

The PGD prevalence rate found corresponds with previous studies, demonstrating that prevalence rates for PGD are especially high in refugees. High prevalence rates of bereavement as well as PGD highlight the need for assessment and specifically tailored treatment of PGD in refugees. PGD goes along with significant psychopathology, which further emphasizes the need for treatment.

## Background

Everyone will eventually experience the loss of a loved person. Loss can be a severe stressor and can lead to painful acute grief symptoms in the bereaved, e.g. yearning or longing for the deceased, intense emotional pain, trouble accepting the loss, or emotional numbness. Grief can have very different manifestations, but symptom intensity typically decreases gradually over time [1]. However, in some individuals grief symptoms are more intense, become chronic, and lead to significant impairment [2]. Prolonged Grief Disorder (PGD) constitutes a syndrome with a unique symptom cluster not covered by other clinical disorders, such as depression, anxiety, or post-traumatic stress disorder (PTSD) [3]. Thus, various experts [3–5] have demanded PGD to be included in the common diagnostic classification systems. In the past years different suggestions regarding criteria have been made [5–7]. One aspect which might account for these different proposals is the fact that there has been a “lack of unanimity in terminology and conceptualization of the disorder” [8], making the distinction between PGD and normal grief, and other mental disorders difficult. Pathological grief itself is described by different terms, i.e. “complicated grief”, “traumatic grief”, “prolonged grief” or “persistent complex bereavement disorder”, which further emphasizes this problem [9]. However, with the increasing body of research in this area, the Diagnostic and Statistical Manual of Mental Disorders (5th ed., DSM-5) [10] eventually included “Persistent Complex Bereavement Disorder” as a temporary research diagnosis calling for further research in this area. In 2018, PGD was included into the International Classifications of Diseases 11 (ICD-11) [4, 11].

As a result of these different conceptualizations, a variety of PGD diagnostic tools exist. The review by Neimeyer and colleagues [12] lists 12 different self-report scales (interviews were not listed at all). In their meta-analysis containing 14 studies, Lundorff and colleagues [13] found 6 different measures. Not only did the different instruments lead to different results when compared to one another (e.g. prevalence of PGD assessed by the Prolonged Grief Disorder Scale (PG-13) = 3.2%, Inventory of Complicated Grief revised (ICG-r) = 9.2%, and ICG = 20.5%), but also within-instrument heterogeneity was rather high, i.e., studies employing the same scale yielded different results. It was discussed that the latter might be a result of different cut-off scores used [13]. Also, prevalence rates for PGD where significantly higher for Western countries (Australia, Denmark, Germany, the Netherlands, United States) in comparison to Eastern countries (China and Japan). The authors offered two explanations for this finding (1) applied methodologies differed or (2) the expression of grief varies significantly across cultures [13].

Research indicates considerable heterogeneity in PGD rates. This will be presented in the following alongside with potential risk factors and comorbid psychopathology.

In a recent systematic review and meta-analysis including 14 studies, the estimated prevalence of PGD in an adult bereaved population was 9.8%, indicating that one out of ten bereaved adults is at risk for PGD [13]. Yet, this review only included studies where bereavement was due to non-violent causes of death. Studies suggest that prevalence rates for PGD are higher if the loss is associated with a violent or man-made cause [14, 15].

Despite the increasing focus on PGD in Western countries, only very few studies have assessed prolonged grief in refugee populations [16] even though traumatic experiences paired with dangers related to the migration process make refugees particularly vulnerable to PGD. Research indicates that the loss of a loved one is significantly associated with psychopathology and low quality of life among refugees [17]. Studies investigating PGD among Bosnian refugees revealed high prevalence rates of PGD, while using different instrument measures (31% used the Core Bereavement Items (CBI) [18]; and 54%, used the Inventory of Complicated Grief (ICG), [19]). As pointed out by a recent study, one factor that might account for the heterogeneity between refugee populations is the significant symptom variation between different cultures [20].

Studies based on Western non-refugee as well as on refugee populations have indicated that being female is a potential risk factor for PGD, as well as a close relationship with the deceased [9, 15, 21–26]. In the study by Morina and colleagues [25] females were six times more likely to suffer from PGD. The fact that care-giving as well as low income and low social economic status have also been associated with a higher risk of PGD, might explain why women in general could be more at risk to develop PGD [9, 23, 24]. Yet, there are also some studies which did not report a significant gender difference [16, 18, 26].

Apart from general socio-demographic aspects, studies have also investigated the relationship between PGD and other mental disorders such as PTSD and depression. Strong correlations between PGD and depression and PGD and PTSD were found in Western non-refugee as well as in refugee populations [18, 25, 27, 28]. Although some symptoms may overlap, those in support of a distinct diagnosis emphasize that nonetheless the different diagnoses can be separated from one another and need different kinds of treatment [3, 4, 29]. In addition to PTSD and depression, PGD is associated with higher levels of somatization and suicide risk [27, 28, 30]. Especially, studies on refugee populations have addressed the importance of somatic symptoms when it comes to bereavement. When asked openly about grief, widows in Nepal reported somatic symptoms [30]. The same applies for Cambodian refugees [31]. PGD in young survivors of the Kosovo war was associated with higher scores of somatic complaints [27]. Consequently, there has been a call for the inclusion of culture-specific somatic items when assessing grief in different cultures [20]. However, several studies have indicated that refugees in general tend to have higher rates of mental disorders, and also that the risk for comorbid somatization in refugees is known to be generally high [32, 33].Taken together, PGD prevalence rates do depend on several factors: the diagnostic instrument used, the subjects’ gender and the country (and the associated culture) where the study was conducted. Furthermore, causes of death as well as relationship with the deceased also have an impact on these estimates, as suggested by Prigerson and colleagues [9, 34]. This was also demonstrated by studies investigating war survivors but not refugees (12.5% assessed by the PG-13 [35]; 34.6% also assessed by the PG-13 [27]; 38.3% assessed by the ICG-r [25]). Finally, PGD appears to often co-occur with other mental disorders [28].

Because prevalence rates of PGD are especially elevated if bereavement is the result of a violent loss, and a high percentage of violent loss can be assumed in war areas, the bereavement of refugees seems to be of particular relevance [17]. As previous research shows, women form an especially vulnerable group in the context of flight, given e.g. the living situation in shared accommodations in the host country and the multiple and complex stressors such as rape or other forms of sexual abuse [36, 37]. Therefore, the aim of this study was to investigate the prevalence of PGD and its association with other psychopathology in female refugees seeking asylum in Germany. The data were collected within the Female Refugee Study [38], which focussed on assessing representative data on the psychosocial situation as well as the access to psychosocial care of refugee women in shared accomodations in Germany.

## Methods

### Design and sampling

This study was part of the “Female Refugee Study” [38] carried out from August until December 2016 in five federal German states. Investigated countries of origin were Syria, Afghanistan, Eritrea, Iran, Iraq, Somalia and Ethiopia based on granting of the status as refugee going along with the likelihood of receiving a positive decision on the asylum application decision. To ensure the data were representative of female refugees in Germany, the Federal Office for Migration and Refugees (BAMF in German) was contacted and statistical data about the distribution of refugees in Germany was obtained. According to these statistics from the BAMF (record date 31.07.2016) a quota was calculated. One hundred participants for the interviews were selected in each of the study sites to represent the composition of nationalities of the German refugee population [38]. Shared accommodations were randomly chosen and contacted. Women there were recruited to be interviewed respecting the quota.

Inclusion criteria were female gender, age of 18 years or older, coming from Syria, Afghanistan, Iraq, Iran, Ethiopia, Somalia or Eritrea, living in shared accommodations and written informed consent. Exclusion criteria were insufficient knowledge of the languages in which questionnaires were available, severe cognitive impairment, acute psychosis and acute suicidality.

### Participants

Originally, the study investigated 106 subjects (i.e. total sample). However, 16 of them did not report any bereavement, thus they were not given the PG-13. Consequently, 90 subjects were interviewed, of which 85 completed the entire interview and these 85 participants were therefore considered in the subsequent analyses (i.e. bereaved subsample).

Mean age of participants was 29.26 years (*SD* = 8.75). The majority of the participants came from Afghanistan (36.14%, *n* = 30) and Syria (32.53%, *n* = 27), followed by Iran (10.84%, *n* = 9), Iraq (9.64%, *n* = 8), Eritrea (6.02%, *n* = 5) and Somalia (2.41%, *n* = 2). 2.41% (*n* = 2) had other citizenships (Tajik, Turkmen), and 2 participants did not provide any information on the country of origin. 33.84 months (*SD* = 57.60) was the mean duration since participants had left their home whereas the mean duration since the application for asylum was 11.05 months (*SD* = 5.50). 42.35% (*n* = 36) of the participants had visited or graduated from school. 20.00% *(n* = 17) had started or finished vocational training or university education. 20.00% (*n* = 17) had not gone to school at all. The vast majority, namely 77.66% (*n* = 66) of the participants had children, and 72.94% (*n* = 62) were married and living together.

### Procedure

Approval was obtained from the ethics committee of the Frankfurt University Hospital (334/16) and from the ethics committee of the Charité Berlin (EA1/117/16). Data collection was completed by trained native speakers who performed either a guided questionnaire assessment, where the interviewer guided the participants through the questionnaires, providing standardized instructions and explanations, or a structured interview (for illiterate women). Most often, structured interviews were conducted, which is in line with Hecker and colleagues [39]. Questionnaires were translated into all languages required (arabic, farsi, somali, tigrinya). For each language, one native speaker translated the questionnaire in the respective language and a different native speaker conducted a back-translation to German that was compared to the original version. This process was repeated if necessary. The translations were discussed with the interpreters before being implemented.

Interviews were carried out by eight trained female interviewers speaking the subject’s native language. All interviewers either had a bachelor’s degree in a related science (psychology, orient studies, or communication) or were advanced in studying psychology or medicine. They received a two-day training in Berlin on interviewing potentially traumatized women. Interviewers also completed another training in Frankfurt to consolidate and expand the previous learned competencies, namely they learned about the specific interview parts, the local structures and contacts. Weekly team meetings and supervision was provided throughout the study, and clinically experienced study coordinators were always available on-call. Telephone calls for spontaneous individual supervision were used frequently.

Randomly chosen shared accommodations were contacted and the study was presented to the management on-site. Female refugees were recruited via information events. Native speakers presented the study and information material during the event. The information sheets and written informed consent were handed out in the respective language. The interviews were conducted at least 24 h after the information to allow time for the women to rethink their willingness to participate.

At any time and without stating reasons the participants had the right to withdraw from participation in the study. Consultations by mother-tongue psychologists or interpreter-aided consultations were offered if required. Assessors were intensively trained to recognize severe psychological impairment and contact the clinicians responsible for the study for further assessment. No women had to be excluded because of the mentioned psychological reasons.

The *Prolonged Grief Disorder Scale (PG-13*) [40] (see also Pfoh G, Rosner R: Deutsche überarbeitete Version des PG-13, unpublished) is an interview to diagnose PGD according to the criteria defined by Prigerson [5, 34]. It consists of 13 items addressing separation distress, cognitive and emotional PGD symptoms plus limitations related to functioning and duration (e.g. “In the past month, how often have you felt yourself longing or yearning for the person you lost?”, “In the past month, how often have you tried to avoid reminders that the person you lost is gone?”). Eleven items are rated on a Likert scale from 1 (not at all) to 5 (several times daily/overwhelmingly) and two items (i.e. duration and impairment) are yes/no questions. In order to receive a PGD diagnosis, five criteria (A-E) have to be met. The first criterion (A) represents the event: the respondent has lost a significant other. In this study the interview was only conducted if criterion A was met. The second criterion (B) is supposed to indicate separation distress. Either item one (longing or yearning for the deceased) or item two (intense feelings of emotional pain) of the PG-13 have to be present on a daily basis (i.e. the respondent has to answer by 4 or 5). Criterion C is a duration criterion; i.e. separation distress must have been present at least for the past 6 months. Criterion D represents cognitive, emotional and behavioural symptoms. PGD-13-items 4–11 represent these symptoms and the respondent has to answer that at least 5 of these symptoms were experienced at least “once a day” or “quite a bit” to meet criteria. Criterion E eventually indicates impairment in social, occupational or other important aspects.

The PG-13 has shown good reliability (Cronbachs α .82), convergent validity (with respect to criteria proposed by Horowitz and colleagues (1997) [7], and discriminant validity with respect to depression (φ = 0.48), PTSD (φ = 0.23) and generalized anxiety disorder (φ = 0.21) coefficients) [5]. A high Cronbachs α of .90 was found in the current study (*M* = 26.61; *SD* = 10.94; *n* = 85). Because the PG-13 can also be scored as continuous measure, [5] item scores (except the items for duration and impairment) were summed up for each participant.

The *Hopkins Symptom Checklist-25* (HSCL-25) [41] is derived from the HSCL [42], and is a screening tool detecting depression and anxiety which has shown high Cronbachs α of .92 in previous research [43]. The checklist consists of two subscales, whereas the reference period is the past month. One is a 10-item scale for anxiety (e.g. “feeling fearful”, “trembling”). The other one is a 15-item scale for depression (e.g. “poor appetite”, “feeling no interest in things”). In the current study Cronbachs α was.95 (*M* = 60.72, *SD* = 18.56; *n* = 67). All items are rated on a Likert scale from 1 (not at all) to 4 (extremely).

The *Somatization Subscale of the Symptom-Checklist-90* [44] records simple physical strain up to functional complaints (e.g. “headaches”, “trouble getting your breath”) with 12 items with high reliability (Cronbachs α .87) [45], which was also confirmed in our study (Cronbachs α .90; *M* = 28.05, *SD* = 11.16; *n* = 78). Items are rated on a five-point Likert scale ranging from “not at all” to “extremely”. The reference period is the last 7 days.

The *Posttraumatic Diagnostic Scale* (PDS/HTQ; [46]) was used to collect previous potentially traumatic events. It includes 25 items that are rated on a 5-point Likert scale ranging from (a) happened to me; (b) witnessed it; (c) having heard of it; (d) part of my job; or (e) neither nor. In our study the internal consistency was very good (Cronbachs α .92). On average 5.34 (*SD* = 4.44) incidents were reported by the participants to have happened to them, and 2.33 (*SD* = 2.96) incidents were reported to have been witnessed on average.

### Data analysis

The data were analyzed using the SPSS Software package (Version 25, IBM Corp., Armonk, NY, USA). Pearson’s correlation was used to investigate relationships between prolonged grief (PG-13) and symptoms related to anxiety and depression (HSCL-25), as well as somatization (SCL subscale) and trauma (PDS/HTQ). To circumvent case-wise deletion, the scale means instead of the sum scores were used after having excluded those respondents who had more than 20% missing values.

Furthermore, binary logistic regression analyses were performed with the PGD diagnostic status as dependent variable and sociodemographic as well as psychopathological outcome measures as explanatory variables.

## Results

9.41% (*n* = 8) of the bereaved subsample were diagnosed with PGD. Thus, prevalence of PGD in the total sample of 106 was 7.55%. The PGD criteria most frequently reported by the participants were bitterness, longing or yearning for the deceased person, and lack of acceptance of the death (see Table 1).

**Table 1:** Agreement to criteria for Prolonged Grief Disorder according to the Prolonged Grief Disorder Scale (PG-13) in the 85 female refugees who reported to have been bereaved

Prolonged Grief Disorder Criteria	*n*	%
Duration of symptoms of at least 6 months	50	58.82
Bitterness over the loss	37	43.53
Longing / yearning for the person lost	33	38.82
Trouble accepting the loss	33	38.82
Intense emotional pain, sorrow, or pangs of grief	32	37.65
Difficulties trusting other people since loss	27	31.76
Feeling confused about role in life, feeling that a part of oneself has died	23	27.06
Functional impairment (in social, occupational, or other areas)	19	22.35
Feeling emotionally numb since loss	16	18.82
Avoiding reminders that the person lost is gone	14	16.47
Difficulties moving on (e.g. making new friends etc.)	14	16.47
Feeling that life is unfulfilling, empty or meaningless	13	15.29
Shocked, stunned or dazed by the loss	9	10.59

Note. Amount or percentage of subjects agreeing to the respective item in the Prolonged Grief Disorder Scale (PG-13)

The PG-13 score displayed significant correlations with anxiety and depression as assessed with the HSCL (*r* = .505, *p* < .001, *n* = 84), and with somatization as assessed with the respective SCL subscale (*r* = .452*, p* < .001, *n* = 83). It was also shown that PGD symptoms significantly correlated with the amount of experienced traumatic events as assessed by the PDS/HTQ (*r* = .310, *p* = .004, *n* = 85), yet no significant association was found with the amount of witnessed trauma (*r* = .167, *p* = .127, *n* = 85).

Table 2 displays frequencies and means of socio-demographic variables as well as psychopathological variables by PGD diagnosis. Also, the results of the logistic regression analysis, which was performed to examine associations between PGD and socio-demographic variables as well as psychopathological measures, are also presented in detail in Table 2. It was decided to begin by looking at the effects of each explanatory variable at a time. Family status and religion were significant socio-demographic predictors of PGD.

**Table 2:** Predicting the likelihood of PGD by sociodemographic and psychopathological factors: results of binary logistic regression analysis

Characteristic	PGD	No PGD	LR (χ^2^)	df	*p*	Nagelkerke R^2^
Country of origin			8.792	6	.186	.214
Afghanistan	4	26				
Syria	0	27				
Iran	1	8				
Iraq	2	6				
Eritrea	1	4				
Somalia	0	2				
Other	0	2				
No	0	14				
Education			4.414	6	.621	.128
No education (did not attend school)	1	16				
Attended / graduated from school	4	32				
Started vocational training	0	3				
Finished vocational training	0	1				
Started / enrolled in university	2	5				
Graduated from university	0	5				
Started vocational training and university	0	1				
Family status			14.147	6	.025*	.368
Single, no relationship	1	8				
Married and living together	3	59				
Married not living together	0	3				
In a relationship	0	2				
Divorced	1	1				
Widowed	1	0				
Partner disappeared, abducted	1	0				
Having children			3.261	1	.071	.084
Yes	8	58				
No	0	14				
Religion			15.211	3	.002*	.353
Islam	3	65				
Other^+^	1	9				
None (unaffiliated)	2	0				
Refused to answer	2	3				
Duration since displacement (in months)	77.50	29.99	2.236	1	.135	.069
Duration since application for asylum (in months)	11.67	11.00	.076	1	.783	.002
Age (years)	27.00	29.49	.679	1	.410	.017
HSCL (mean)	2.900	2.416	3.054	1	.079	.077
SCL Somatization (mean)	3.094	2.248	5.589	1	.018*	.139
Number of experienced trauma	9.250	4.935	5.596	1	.018*	.137
Number of witnessed trauma	3.875	2.169	2.058	1	.151	.052

*PGD* Participants with PGD, *no PGD* Participants without PGD, *LR* Likelihood ratio. Categorical variables are reported as frequencies. Other metric data are reported as means. * *p* < .05 ^+^ various categories (e.g. Christian, Judaism, Hinduism etc.) were provided, but were summarize as ‘other’ since this category applied to a minority only

Somatization and the number of experienced traumatic events were significant predictors of PGD, which thereby confirms other studies that found somatic symptoms as well as exposure to trauma being associated with PGD [14, 15, 20]. There was a trend for having children as well as depression and anxiety assessed by the HSCL-25 to also be associated with PGD.

As a final step, all explanatory variables leading to significant effects in the separate logistic regression analyses (family status, religion, mean score of SCL somatization subscale and number of experienced trauma) were put into one logistic regression model to test for the effect of all explanatory variables simultaneously. A significant effect could be found, χ^2^_(10)_ = 19.069, *p* = .039, Nagelkerke R^2^ = .476 (*n* = 79).

## Discussion

The first aim of the present study was to investigate the prevalence of PGD in female refugees seeking asylum in Germany who were living in shared accommodations. A substantially high percentage of 84.91% of the total sample reported bereavement, which is in line with the study provided by Hengst and colleagues [17]. A total of 9.41% (*n* = 8/85) of the bereaved subsample was diagnosed with PGD according to the PG-13. This prevalence rate corresponds with the one found in a current meta-analysis for PGD in bereaved subjects [13]. Yet, as already mentioned, this meta-analysis focused on natural deaths only, and therefore did not include studies where suicide, murder or violence caused bereavement. The meta-analysis by Lundorff and colleagues [13] displayed variations in prevalence rates depending on the diagnostic tool used for assessment. When compared only to the results provided by studies which also used the PG-13, the prevalence rate in our study exceeds the reported rates so far (i.e. 9.41% compared to 3.2%). This is consistent with the findings provided by other studies, generally implying that traumatic loss rather tends to elicit PGD [14, 17], which is assumed to apply to the present sample.

Looking at studies reporting an effect of female gender on grief, a distinction has to be made between studies simply reporting group differences in scale means (not providing information on the true prevalence rate) and studies reporting prevalence rates. Most of the time prevalence rates are reported for the complete sample. However, for non-refugee samples two studies reported prevalence rates for women: in their large German sample (*n* = 805 women) Kersting and colleagues reported a prevalence rate of 8.2% (assessed with the ICG) [24]. In contrast, Fujisawa and colleagues only found a rate of 3.4% (assessed with the Brief Grief Questionnaire) in a total sample of 564 female participants in Japan [22]. Due to the large number of participants both samples can be regarded representative. The prevalence rate obtained in our study exceeds these findings. In addition to these non-refugee findings, two studies assessing PGD in refugees reported high prevalence rates in females. Both, the study by Schaal and colleagues [35] and Morina and colleagues [25] employed the PG-13 and obtained prevalence rates of 12.5 and 65%, respectively. First, as can be seen, the rates are very different. Yet, so is the cultural context and this might be one factor accounting for this finding. Either PGD symptoms differ significantly between cultures or diagnostic tools are irregularly sensitive towards different cultures [20]. The study of Schaal and colleagues investigated widows in Rwanda, who had lost their husband due to genocide [35], whereas the Morina et al. study investigated bereaved Kosovar civilian war survivors [25]. Second, both samples were rather small (40 in Schaal et al. and 20 participants in Morina et al.) which makes it unclear whether these studies have been representative for the respective population.

As can be seen, the reported prevalence rates of PGD are subject to cultural context and representativeness of the sample. To our knowledge there are no studies on PGD in Middle Eastern refugees, which makes it difficult to fully compare our results with the ones mentioned above. Yet, our study is able to close an important gap, as it is based on a larger sample size than other studies on PGD in refugees. Cultural implications will be discussed further in the Limitations section.

Another aspect which might have influenced our prevalence rate is the circumstance that as participation was voluntarily, we assume that women with strong psychological strain were not interested in participating in our study.

The second aim of this study was to associate PGD with other psychopathology. This study showed significant associations between PGD severity and symptoms of somatization, number of experienced trauma, anxiety, and depression, implying that PGD is accompanied with significant psychopathology, as was already reported by others [2, 6, 14, 15, 18, 20, 27]. The finding that somatization can be associated with PGD is of special interest, since it has been outlined that somatic symptoms are often reported along with grief in specific cultures [20, 27, 31]. Yet, standard grief measures, which have been validated by Western non-refugee populations mostly, do not assess these kinds of symptoms, which makes it even more important to assess them at least separately [20, 47].

In summary, this study demonstrates that PGD should be considered when assessing psychological disorders among this group and when planning interventions. This means, when treating refugees, standard diagnostic evaluations should always also include questions on important losses [48]. Otherwise, without assessing possible bereavement, co-occuring psychopathological symptoms actually associated with PGD, as for example somatic symptoms, might be interpreted differently, eventually resulting in wrong diagnosis and unhelpful treatment [20]. Studies on the treatment of PGD have shown that specific treatment of PGD, e.g., a combination of cognitive restructuring and exposure therapy, can be very effective [49, 50]. Yet, these studies have been conducted on non-refugee populations. Other promising approaches could be writing exercises as already used by Kalantari and colleagues in bereaved Afghani refugees [51] or an adaption of culturally adapted CBT as seen in Hinton and colleagues [52].

Since there were only very few cases in the bereaved subsample qualifying for PGD diagnosis (*n* = 8 vs. *n* = 77 not qualifying for PGD diagnosis) the results of the binary logistic regression should be interpreted with caution. First, the analysis confirmed associations between somatization and number of experienced trauma by revealing significant effects for both. It can be assumed that women suffering from somatization or having experienced a higher amount of traumatic events have more difficulties with bereavement as former research also shows this association [14, 15, 20]. Future studies should continue to investigate the role of these aspects.

In addition to that, religion and family status were also significant predictors. Yet, the fact that these categorical variables have more than two categories makes it difficult to interpret the data. Looking at both of these variables, it can be seen that cases are far from being distributed equally (e.g. 76.47% of our participants were Muslims, and 72.94% were married and living together). This unequal distribution of participants across categories makes it difficult to interpret and, consequently, to discuss these results. However, usually religion and social support, with the latter possibly to be provided by relatives, are considered protective factors after bereavement [53].

Having children and the results of the HSCL-25 failed to reach significance as predictors in the single models, but both indicated a trend. Again, having children was unequally distributed as a majority of the participants reported having children (*n* = 66, i.e. 77.66%). All individuals suffering from PGD had children. The finding that depression and anxiety trend to predict PGD corresponds with previous research results, where this kind of psychopathology had also been associated with PGD in different contexts [25, 27, 28].

## Limitations

We captured PGD prevalence using a rigorous diagnostic tool based on the consensus criteria offered by Prigerson [5], which were very likely to be proposed ICD-11 criteria at time when this study was designed and carried out in 2016. The ICD-11 criteria proposed eventually in June 2018 differ slightly from the ones formulated by Prigerson and colleagues [5, 11], e.g. the symptoms listed in both proposals are labelled differently, but most of them can be found in both. According to ICD-11 longing or persistent preoccupation needs to be accompanied by at least one additional symptom [11]. In contrast, the criteria offered by Prigerson and colleagues demand at least five additional symptoms along with separation distress [5]. A direct comparison of both conceptualizations and detailed analysis can be found elsewhere [54]. It is likely that future studies will resort to the ICD-11 criteria resulting in higher prevalence rates.

Results are further limited by the rather small sample size. Since the objective of the main study [38] was to investigate the psychosocial situation of female refugees, not the clinical diagnoses, we collected data allowing the assessment of traumatic experiences but not the clinical diagnosis of PTSD. This prevented the investigation of the relationship of clinical PTSD, or in fact any other mental disorder, and PGD in the current study. Consequently, future studies should not only investigate the prevalence of PGD in large and representative samples of refugees but also assess comorbid mental disorders when doing so.

One further limitation of this study is represented by the lack of general information on the causes of death, time since loss as well as on kinship with the deceased. All of these variables have demonstrated to be of crucial importance, as they represent potential risk factors when it comes to the development of PGD and would have added important information for predicting PGD symptom severity [15].

Also, different post-migration stressors which could also be a factor contributing to the maintenance of mental health problems such as PGD in our sample have not been recorded. As former research shows, the post-migration environment is predictive of mental health outcomes in refugees [55, 56]. For example, length of time in country prior to receiving services, social and economic strain, and alienation [57, 58] were found to contribute to psychopathology. Insecure asylum status and severe financial difficulties post migration are also important for explaining mental disorders [59]. Furthermore, refugees are more likely to be able to experience a natural process of mental healing when post-migration stressors are absent [56]. Therefore, post-migration stress is an important consideration for upcoming research about PGD in refugees.

One final important aspect limiting the utility and validity of the results is the fact that a diagnostic tool was employed which has been validated in a Western population [47]. As it has been outlined by Bolton and Tang [60] culture specific questions cannot be adapted to other cultures easily, thus resulting in a lack of content validity. One option to face this problem could be the development of individual questionnaires, assessing the ability to do certain tasks as in Bolton and Tang [60] or including local idiom and culturally bound symptomatology as proposed by Killikelly et al. [20]. Also, the use of different answering formats, e.g., non-verbal response cards [60] might benefit illiterate individuals. Additionally, cultures have very different norms when it comes to mourning, suggesting that mourning for a longer period of time may be considered acceptable and normal in one culture but not in another [47]. Finally, knowledge of the patient’s culture will benefit clinician-patient interaction and patient satisfaction in general, creating a major impact on mental health and well-being [61, 62].

## Conclusion

The present study is the first study to present prevalence rates of PGD in refugees in a Middle Eastern refugee sample. The obtained prevalence rate and associations with other psychopathology demonstrate that PGD is a relevant concern in refugees, which should be considered during the processes of diagnostic assessment and also when it comes to planning interventions.

Yet, tools for diagnostic assessment either have to be newly developed in order to be sensitive towards cultural peculiarities or have to be adapted at least. The same applies for the dissemination of specific interventions.

## Abbreviations

5th ed., DSM-5: Diagnostic and Statistical Manual of Mental Disorders
CBI: Core Bereavement Items
HSCL-25: Hopkins Symptom Checklist-25
ICD-11: International Classifications of Diseases 11
ICG: Inventory of Complicated Grief
ICG-r: Inventory of Complicated Grief revised
PG-13: Prolonged Grief Disorder Scale
PGD: Prolonged Grief disorder
PTSD: Post-traumatic stress disorder
